# Patterns of Legume Purchases and Consumption in the United States

**DOI:** 10.3389/fnut.2021.732237

**Published:** 2021-10-12

**Authors:** Richard D. Semba, Nihaal Rahman, Shutong Du, Rebecca Ramsing, Valerie Sullivan, Elizabeth Nussbaumer, David Love, Martin W. Bloem

**Affiliations:** ^1^Johns Hopkins Center for a Livable Future, Johns Hopkins University, Baltimore, MD, United States; ^2^Wilmer Eye Institute, Johns Hopkins University School of Medicine, Baltimore, MD, United States; ^3^Department of Epidemiology, Johns Hopkins Bloomberg School of Public Health, Baltimore, MD, United States; ^4^Welch Center for Prevention, Epidemiology, and Clinical Research, Johns Hopkins University, Baltimore, MD, United States; ^5^Department of Environmental Health and Engineering, Johns Hopkins Bloomberg School of Public Health, Baltimore, MD, United States

**Keywords:** beans, climate change, consumer, diet, greenhouse gas, legume

## Abstract

**Background:** Legumes are an inexpensive, healthy source of protein, fiber, and micronutrients, have low greenhouse gas and water footprints, and enrich soil through nitrogen fixation. Although higher legume consumption is recommended under US dietary guidelines, legumes currently comprise only a minor part of the US diet.

**Objectives:** To characterize the types of legumes most commonly purchased by US consumers and patterns of legume purchases by state and region, seasonality of legume purchases, and to characterize adults that have a higher intake of legumes.

**Methods:** We examined grocery market, chain supermarket, big box and club stores, Walmart, military commissary, and dollar store retail scanner data from Nielsen collected 2017–2019 and dietary intake from the National Health and Nutrition Examination Survey (NHANES), 2017–2018.

**Results:** The five leading types of legumes purchased in the US were pinto bean, black bean, kidney bean, lima bean, and chickpea. The mean annual per capita expenditure on legumes based on grocery purchases was $4.76 during 2017–2019. The annual per capita expenditure on legumes varied greatly by state with highest expenditure in Louisiana, South Carolina, Florida, Alabama, Mississippi, and lowest expenditure in Washington, New York, and Wisconsin. There were large regional differences in the most commonly purchased legumes. Of 4,741 adults who participated in the 24-h dietary recall in NHANES, 2017–2018, 20.5% reported eating any legumes in the previous 24 h. Those who consumed legumes were more likely to be Hispanic, with a higher education level, with a larger household size (all *P* < 0.05), but were not different by age, gender, or income level compared to those who did not consume legumes.

**Conclusion:** Although legumes are inexpensive, healthy, and a sustainable source of protein, per capita legume intake remains low in the US and below US dietary guidelines. Further insight is needed into barriers to legume consumption in the US.

## Introduction

Legumes have been a basic part of the human diet since the advent of agriculture and development of civilization in the Middle East, Asia, the Americas, and Europe (1). Legumes are an important source of protein, dietary fiber, complex carbohydrates, iron, zinc, B complex vitamins, and essential amino acids, and are practically free of saturated fats (2). The major sources of dietary protein are meat, poultry, fish, eggs, dairy products, legumes, and nuts. Of these protein sources, legumes are healthy, relatively inexpensive, and widely available. Legumes have low greenhouse gas (GHG) and water footprints compared to animal meat production, enrich soil through nitrogen fixation, and are an environmentally sustainable source of dietary protein (3). However, the average consumption of legumes worldwide remains low at 21 g/person/day compared to 112 g/person/day for meat (3). The average per capita consumption of legumes in the US is only 9.3 g/day (4).

For the purpose of this paper, legumes are defined as edible seeds of the family Leguminosae (3, 5, 6) and include species that are commonly available in US retail stores such as common bean (*Phaseolus vulgaris*), chickpea (*Cicer arietinum*), lentil (*Lens culinaris*), fava bean (*Vicia faba*), mung bean (*Vigna radiata*), soybean (*Glycine max*), common pea (*Pisum sativum*), pigeonpea (*Cajanus cajan*), black-eyed pea (*Vigna unguiculata*), and lima bean (*Phaseolus lunatus*). The numerous cultivars of *Phaseolus vulgaris* include kidney bean, black bean, cannellini bean, pinto bean, pink bean, great northern bean, cranberry bean, mayocoba bean, white bean, navy bean, yellow bean, purple bean, and turtle bean.

Plant-forward diets, such as the planetary health diet recommended by the EAT-Lancet Commission, emphasize a shift from animal-source protein to greater consumption of legumes and nuts for optimal health and sustainability (7). Higher plant protein intake has been associated with lower all-cause and cardiovascular mortality (8). The patterns of legumes consumption in the US have not been well characterized and are important in assessing dietary habits. Legume consumption has been historically low in US adults (9, 10). Little is known about legume purchases from the retail sector except for proprietary industry data that is mainly used for marketing decisions; retail sector data is not widely used by the health community besides sugar sweetened beverages and tobacco (11, 12). In addition, the dietary consumption of legumes has not been well characterized on the state level. The National Health and Nutrition Examination Survey (NHANES), the most commonly utilized dataset on dietary consumption in the U.S. population, is generally restricted below the national level (13).

The specific aims of this study were to characterize: (1) the types of legumes that are most commonly purchased by US consumers, (2) legume purchases by state and region (Northeast, Midwest, South, and West) of the US, (3) legume purchases by season, and (4) adults with a high vs. low dietary consumption of legumes. To answer these aims, we analyzed two independent sources of data, grocery market and other retail scanner data and dietary intake data from the National Health and Nutrition Examination Survey (NHANES), 2017–2018.

## Methods

Purchases of legumes were based upon retail scanner data collected by Nielsen (eXtended All Outlet Combined, xAOC, product, New York, NY) during a 3-year period from January 1, 2017 to December 31, 2019. Data on retail sales volume and revenue were collected by Nielsen from participating retail brands in 31 states. The sample consisted of 237 store brands and 109,695 individual store locations, including grocery, convenience, drug, club, big box, military, pet, and dollar stores, defined in this analysis as “retail.” The xAOC data include all purchase data combined from these retail stores. Nielsen uses a proprietary model to estimate total retail sales by state and for the entire U.S. The Nielsen data is nationally representative of U.S. food retail sales, as it includes 90% of grocery stores. Nielsen makes projections for the remaining stores, except Costco.

Data were collected for the annual sales of legumes at the universal product code (UPC) level. The UPC provides a unique number for each product and is much like a barcode. The dataset does not report UPC-level sales by store brand, so for example, we were not able to separate out sales from grocery stores vs. big box stores, or one grocery store chain from another. Data were deflated using the Consumer Price Index (CPI-U series, non-seasonally adjusted) and pegged to 2019 for annual deflation. Regional price indices were used for state-level data.

The food items included in this analysis were: pinto bean, black bean, kidney bean, lima bean, chickpea, great northern bean, black-eyed pea, soybean, “value added” bean, lentil, cannellini bean, pigeon pea, navy bean, mayocoba bean, split pea (common pea), pink bean, white bean, cranberry bean, remaining dried bean (e.g., calypso bean, white pea bean, Jacob's cattle bean, and orca bean), yellow bean, fava bean, turtle bean, mixed bean, mung bean, dried bean, and purple bean, in the form of dry, canned, or frozen. “Value added” beans consisted of beans that have been prepared or “precooked” to save preparation time. Although peanut (*Arachis hypogaea*) is a member of the family Leguminosae, it is usually grouped with nuts in dietary analyses and is not included in the present analysis. Green beans, French beans, green peas, and pole beans, fresh or frozen, were considered vegetables and were excluded from the present analysis. Tofu, prepared foods such as burritos and salsas, canned products mixed with legumes, such as beef stews, chili with beans, bean soups, and meat analogs, were not included in the present analysis of Nielsen data. The average national revenue generated by each type of legume was calculated based upon the average revenue generated from 2017 to 2019 for each food item using the “dollars” variable.

The average revenue generated within each of 31 states was calculated by averaging the total “dollars” amount for each food item. Average per capita spending on legumes by state was calculated for each food item by dividing the average revenue in each state by the respective average population from US census population data (14) in the 3-year period. Regional average per capita spending on legumes was calculated by dividing the 31 states into four regions: Northeast (Connecticut, Massachusetts, New Jersey, New York, Pennsylvania), Midwest (Illinois, Indiana, Kansas, Michigan, Missouri, Minnesota, Ohio, Wisconsin), South (Alabama, Florida, Georgia, Kentucky, Louisiana, Maryland, Mississippi, North Carolina, South Carolina, Tennessee, Texas, Virginia), and West (Arizona, California, Colorado, Nevada, Oregon, Washington). To examine seasonality in legume purchases, we divided the calendar months into spring (March, April, May), summer (June, July, August), fall (September, October, November), and winter (December, January, February) and used the annual average for 2017–2019.

In order to characterize legume consumption among US adults, ≥20 years, we examined data from a single 24-h dietary recall interview conducted during the NHANES, 2017–2018 (15). A cut-off of ≥20 years was used to be consistent with NHANES age categories in reporting results. Standardized dietary recall interviews were conducted in a private room of the Mobile Examination Center by trained interviewers who were fluent in Spanish and English. The original sample consisted of 9,254 individuals who were interviewed, of whom 4,513 were excluded (3,685 < 20 years of age, 828 with missing or unreliable dietary recall data), giving a final sample of 4,741. We included people who provided at least one 1 (day 1) 24-h recall that was deemed to be complete and reliable by the Food Surveys Research Group. A second 24-h recall was not completed by 12.7% of adults. Among those reporting legume consumption in the last 24 h, we calculated the percentage of legume consumption by breakfast, lunch, dinner, snack or drinks, using the definition of Kant & Graubard (16) and the average legume intake at these occasions.

Consumption of legumes was based upon the variable DR1I_PF_LEGUMES (mature or dried beans, peas, and lentils, in ounce-equivalents) (17) from the United States Department of Agriculture (USDA) Food Patterns Equivalents Database (FPED) (18). FPED converts foods and beverages consumed in the Food and Nutrient Database for Dietary Studies (FNDDS) 2017–2018 to 37 USDA Food Patterns components. The FNDDS provides the recipes and nutrient values for foods and beverages reported in the dietary intake component of NHANES. Consumers were defined by any legume intake >0 ounce-equivalents. Ratio of family income to poverty variable was categorized to three groups. Mexican-American and Other Hispanic variables were combined to create the variable Hispanic. In the comparison of adults with and without legume consumption in the 24-h dietary recall, Pearson's chi-squared test was used for categorical variables and Student's *t*-test was used for continuous variables. Taylor series linearization was used to calculate standard errors. Sample weights, strata, and primary sampling unit variables were specified using survey procedures in Stata to account for the complex, multistage, probability sampling strategy of NHANES and obtain nationally-representative estimates. Dietary day 1 sample weight was used to adjust the NHANES data.

## Results

**Figure 1:**
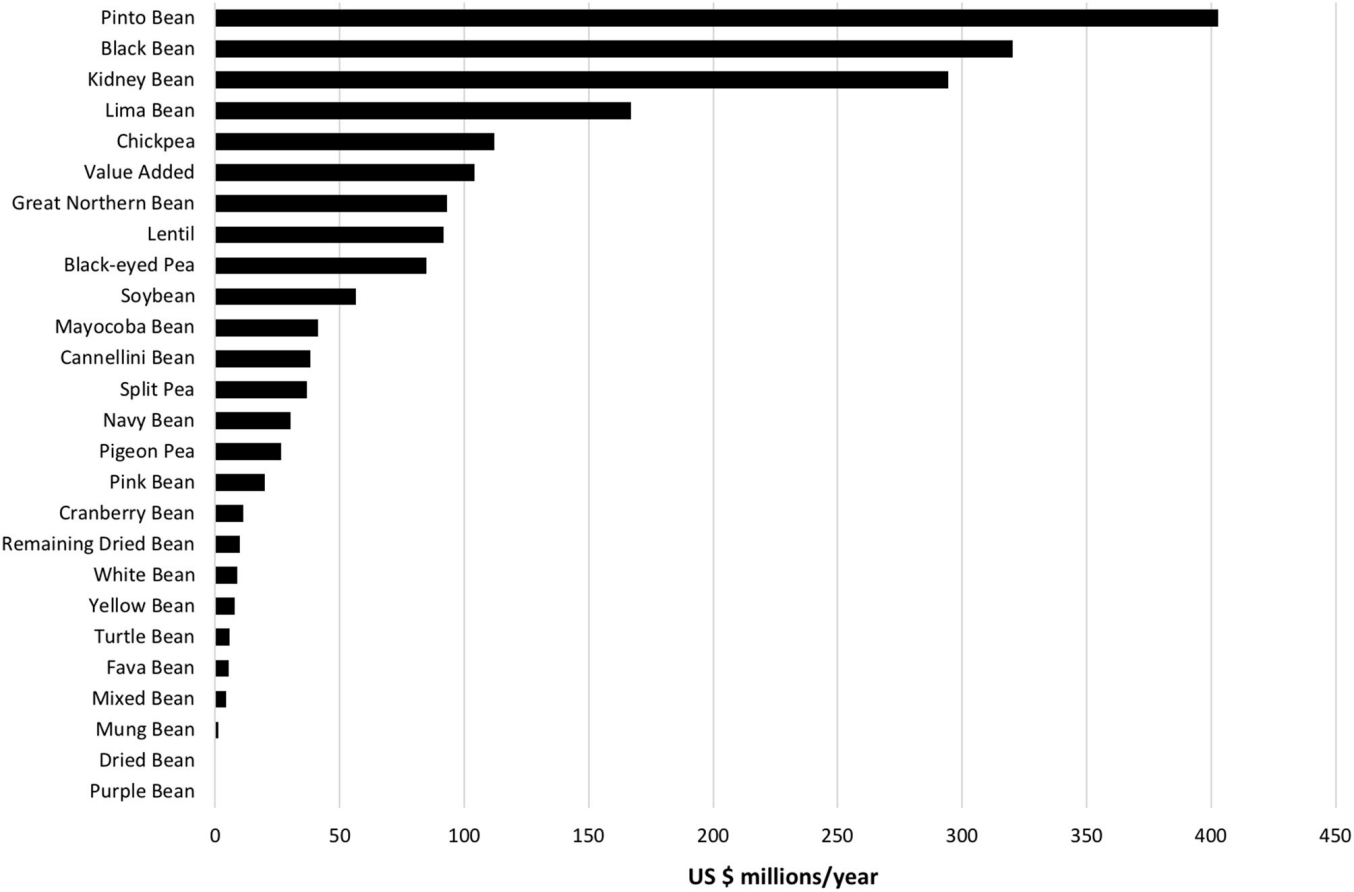
Annual expenditure on types of legumes in the US based upon Nielsen retail grocery scanning data, average over 2017–2019 period.

The type of legume or legume category that accounted for the top 10 grocery store purchases by cost in the US were pinto bean, black bean, kidney bean, lima bean, chickpea, “value added” bean, great northern bean, lentil, and black-eyed pea. Total expenditure per year by type of legume or legume category, averaged over the 3-year period 2017–2019, is shown in Figure 1. Over US $250 million per year was spent on pinto bean, black bean, and kidney bean, respectively. Consumers spent about US $100–200 million on lima bean, chickpea, and “value added” bean, respectively, with lesser amounts spent on the other types or categories of legumes (Figure 1).

**Figure 2:**
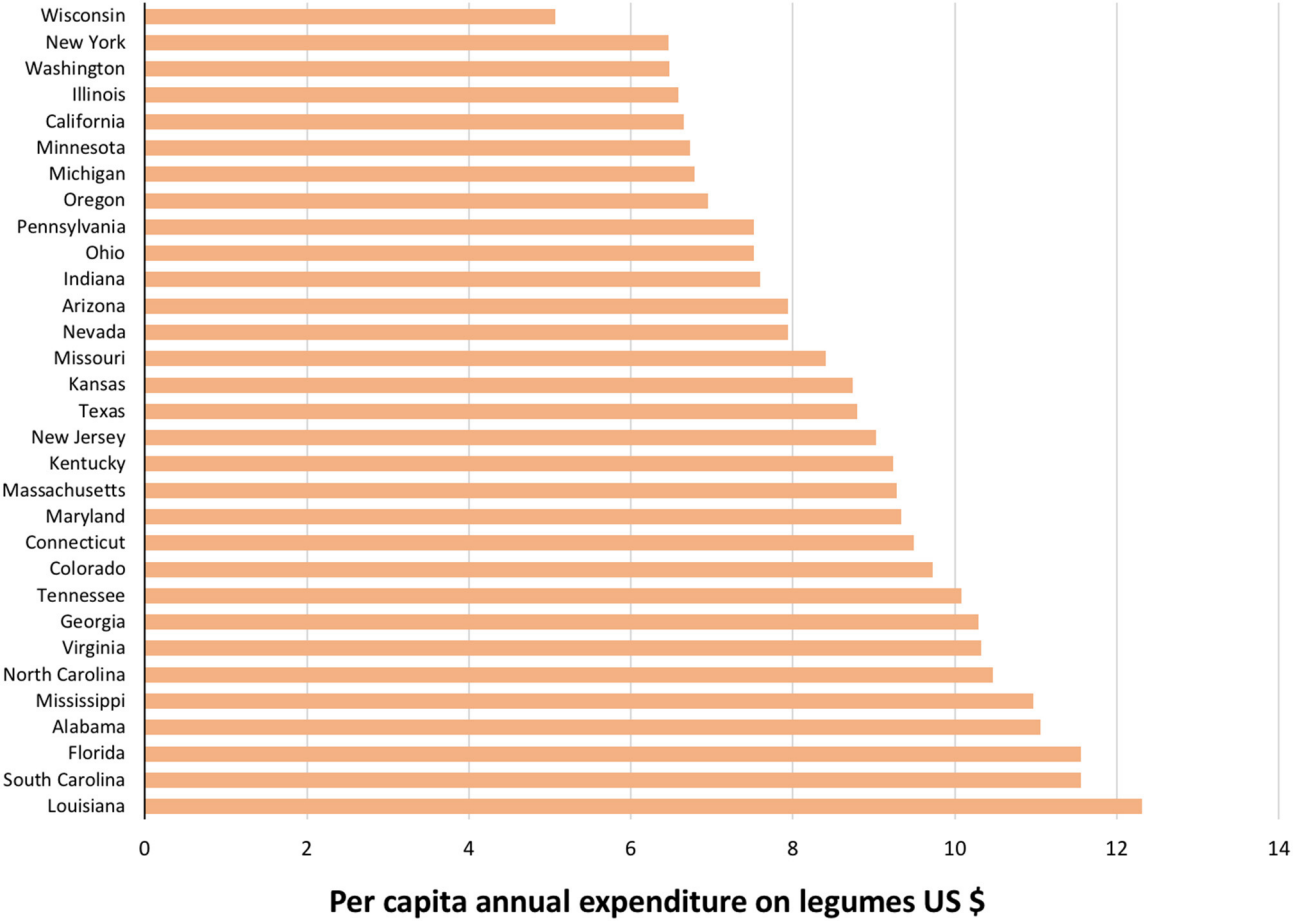
Per capita expenditure on legumes by state based upon Nielsen retail grocery scanning data, average over 2017–2019 period.

The overall mean (standard deviation) annual per capita expenditure in the US during 2017–2019 was $4.76 (1.13), based upon total legume expenditures in 31 states divided by the population. The annual per capita expenditure on legumes varied greatly by state (Figure 2). The three states with the highest expenditure were Louisiana, South Carolina, and Florida. The annual per capita expenditure on legumes was lowest in Wisconsin, New York, and Washington.

**Figure 3:**
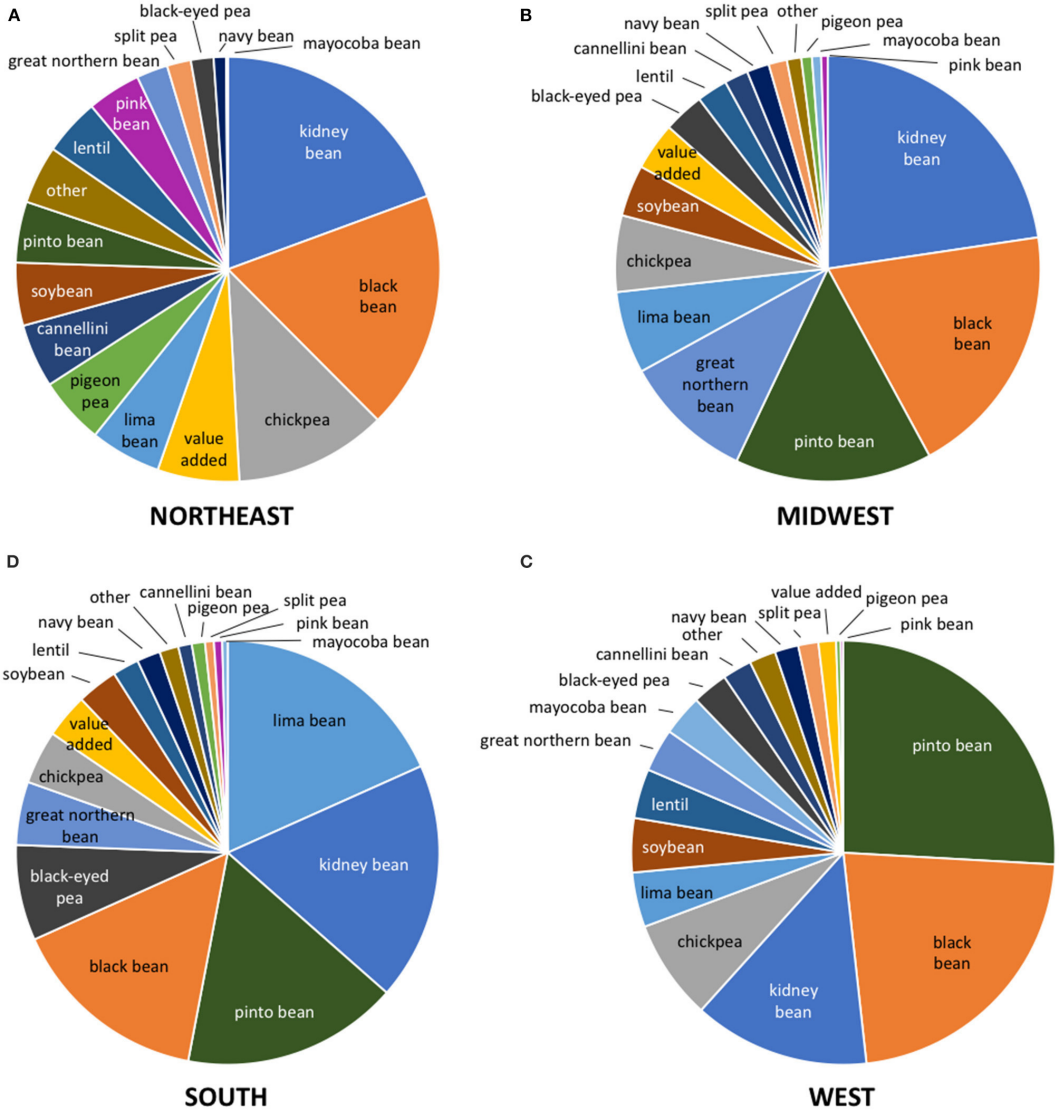
Per capita expenditure on type of legume by region of US: **(A)** Northeast, **(B)** Midwest, **(C)** West, **(D)** South.

There were also regional differences in the types of legumes that were purchased (Figure 3). In the Northeast, kidney bean, black bean, and chickpea accounted for the top three types of legume purchases. Kidney bean, black bean, and pinto bean were the top three types of legume most commonly purchased in the Midwest. Lima bean, kidney bean, and pinto bean were the top three most commonly purchased legumes in the South, whereas pinto bean, black bean, and kidney bean were most commonly purchased in the West. Average annual expenditure and per capita expenditure on the types of legumes in each state are presented in detail in Supplementary Table 1.

**Figure 4:**
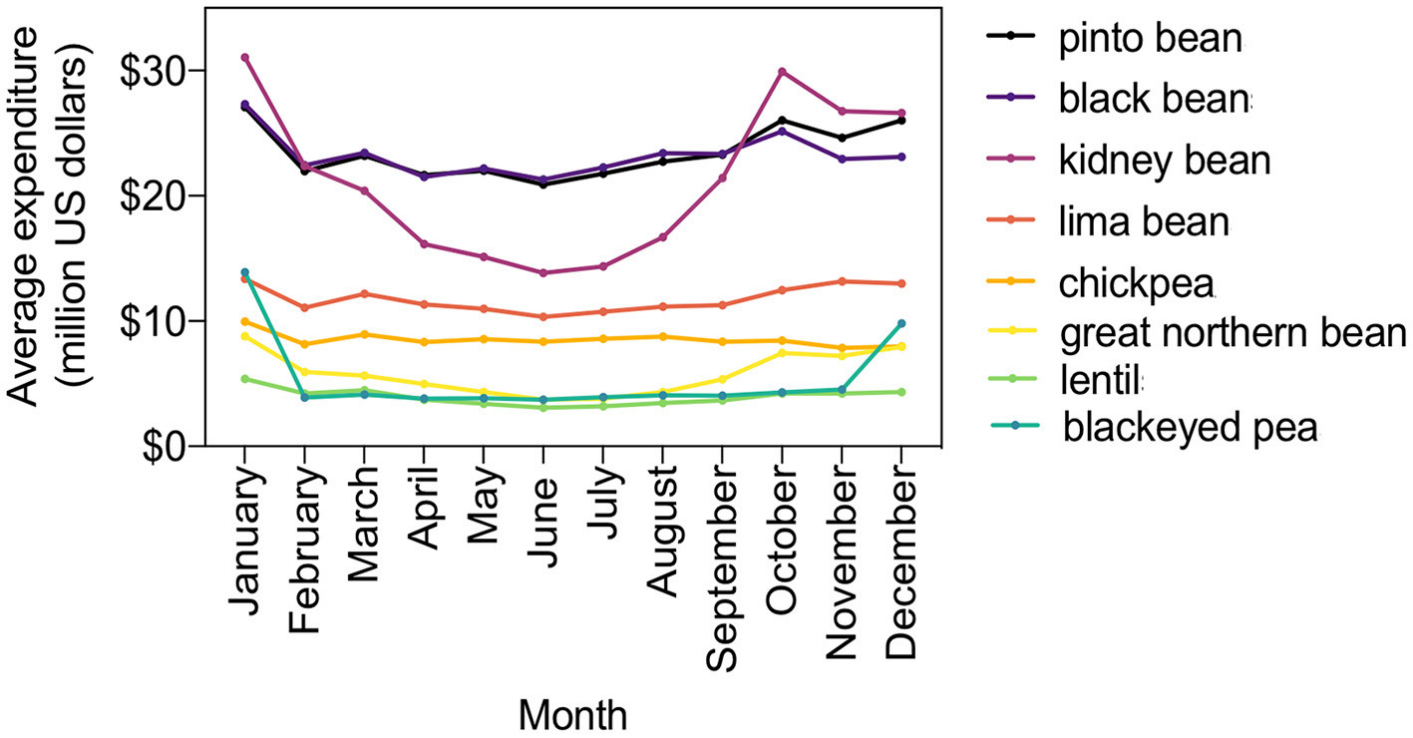
Monthly expenditures on the top eight legumes in the US based upon Nielsen retail grocery scanning data, average over 2017–2019 period.

Legume purchases were lowest during the summer and peaked during the winter. The total amounts of expenditures on legumes in spring, summer, fall, and winter were US $353, 328, 400, and 433 million, respectively. The amount spent per month on the eight leading types of legumes are shown in Figure 4. The highest expenditures on legumes were made in the month of December.

Based upon the 24-h dietary recall in NHANES, 2017–2018, 20.5% of US adults consumed any legumes in the previous 24 h (Table 1). There were no significant differences between those who did or did not report consuming legumes by age, gender, or income. Among individuals that recalled eating legumes, those with higher compared with lower intake levels were more likely to be Hispanic, with a higher education level, and with a larger household size (all *P* < 0.05; Table 1). Legume intakes of 990 adults who reported consuming legumes in the previous 24 h are shown in Table 2. Legume intake was significantly higher among men compared with women (*P* < 0.001), Hispanics compared with other race categories (*P* = 0.007), those with less than a high school education (*P* = 0.005), and those with a lower income to poverty level ratio (*P* = 0.004). The location and occasion (meal or snack) of legume consumption among those who reported consuming legumes in the last 24 h are shown in Table 3. The highest proportion of legumes are consumed at dinner and a third of legumes are consumed away from home.

**Table 1:** Characteristics of adults (≥20 y) who reported consuming legumes on a single dietary recall in the National Health and Nutrition Examination Survey, 2017–2018.

**Characteristic^a^**		**Legume intake last 24 h**		***P^b^***
		**No**	**Yes**	
		***n* = 3,751**	***n* = 990**	
Age, y		48.7 (0.8)	47.1 (0.7)	0.20
Gender, %	Male	48.1	47.7	0.81
	Female	51.9	52.3	
Race^c^, %	White	64.1	54.0	<0.001
	Black	12.6	7.6	
	Asian	5.3	8.2	
	Hispanic	13.0	26.7	
	Other, or multiple races	5.0	3.5	
Education^d^, %	< High school	10.1	13.2	0.01
	High school graduate	29.2	23.9	
	>High school	60.7	62.9	
Income to poverty level ratio^e^, %	<131% Poverty	21.3	18.6	0.23
	131–350% Poverty	36.0	34.1	
	>350% Poverty	42.8	47.3	
Number of household members		3.0 (0.06)	3.3 (0.09)	0.005

a *Characteristics are reported as mean (standard error) for continuous variables and percentages for categorical variables*.

b *P-values calculated from Pearson's chi-squared test for categorical variables and t-test for continuous variables*.

c *Non-Hispanic white, non-Hispanic black, non-Hispanic Asian*.

d *Missing n = 8 values for education*.

e *Missing n = 558 values for poverty income ratio*.

**Table 2:** Legume intake among 990 adults in NHANES who reported consuming legumes in the last 24 h.

**Characteristic^a^**		**Legume intake as protein (oz) in the last 24 h**	***P* ^b^**
Total		2.35 (0.13)	N/A
Gender	Male	2.79 (0.17)	<0.001
	Female	1.95 (0.15)	
Race^c^	White	1.94 (0.13)	0.007
	Black	2.33 (0.27)	
	Asian	2.60 (0.42)	
	Hispanic	3.05 (0.19)	
	Other, or multiple races	2.89 (0.79)	
Education^d^	< High school	3.36 (0.23)	0.005
	High school graduate	2.26 (0.19)	
	>High school	2.17 (0.16)	
Income to poverty level ratio^e^	<131% Poverty	2.96 (0.22)	0.004
	131–350% Poverty	2.45 (0.16)	
	>350% Poverty	1.86 (0.20)	

a *Characteristics are reported as mean (standard error)*.

b *P-values calculated from adjusted Wald test*.

c *Non-Hispanic white, non-Hispanic black, non-Hispanic Asian*.

d *Missing n = 1 value for education*.

e *Missing n = 150 values for poverty income ratio*.

**Table 3:** Percentage and the amount of legume intake by eating occasion and location among 990 adults in NHANES who reported consuming legumes in the last 24 h.

		**Proportion of Total Intake (%)**	**Average intake(protein oz-equivalents)^a^**
Eating Occasion	Breakfast	13.6	0.38 (0.04)
	Lunch	30.7	0.68 (0.08)
	Dinner	47.2	1.18 (0.10)
	Snack or drinks	8.40	0.10 (0.01)
Location	At home	67.0	1.69 (0.10)
	Away from home	33.0	0.66 (0.09)

a *Mean (standard error)*.

## Discussion

The present study shows that of the different species of legumes, cultivars of the common bean, *P. vulgaris*, dominated legume purchases in the US, followed by lima bean, chickpea, black-eyed pea, soybean, and lentil. There were large regional differences in grocery store purchases of legumes in the US. Legume purchases in the South were about 50% higher than other regions in the US. Per capita legume purchases in Louisiana were more than two-fold higher than in Wisconsin. There were also regional differences in the types of legumes that were purchased. Lima bean was the leading type of legume purchased in the South, which may reflect the legacy of the American Indians who originally cultivated lima bean in this region (19, 20). Lima bean became a preferred legume in regional recipes in the South (21, 22) and is currently consumed in dishes such as succotash, Kentucky burgoo, and Southern butter beans. In the West, pinto bean was the leading type of legume purchase. Legumes, notably pinto beans, are a common item in the traditional western meal (23). Kidney bean and black bean were the leading types of legumes purchased in the Midwest and Northeast regions.

About one-fifth of US adults in NHANES reported consuming legumes by 24 h dietary recall. Cultivation of *P. vulgaris* originated in the Andean region and Mesoamerica, and legumes are a traditional element of Latin American cuisine (3). Those reporting legume intake were more likely to be Hispanic. Higher legume consumption has been previously reported elsewhere among Hispanic adults, especially among those who are less acculturated (24, 25). Although legumes have a traditional reputation as “poor man's meat” (1), there were no differences among those with and without legume consumption by income, and those reporting eating legumes had a significantly higher level of education. However, among those who consumed legumes in the previous 24 h, the amount of legumes consumed was higher among those who were Hispanic, with lower education, and lower socioeconomic status. A previous study of legume consumption in NHANES showed that of those reporting legume consumption, legumes were mainly consumed as a side dish (beans alone or combined with another vegetable) or main dish (combined with rice/meat/stew/chili), and less often as soups, dips, or salads (10).

We used 24-h recall dietary data from NHANES for the present analysis. A single 24-h recall per person is regarded as sufficient to estimate population mean dietary intakes, under the assumption that a 24-h recall is an unbiased measure of true intake and that collection days are representative of the seasons and days of the week (26). Our goal was to estimate mean intakes in the population overall and in important subgroups, rather than describe the distribution of individuals' usual intakes for comparison against a reference standard. Therefore, a single 24-h recall is adequate and avoids excluding the possibly nonrandom 12.7% of adults who did not complete a second 24-h recall.

The present study is limited to a cross-sectional analysis and does not show how legume purchases have changed over time. The consumption of legumes worldwide, including the US, has been flat (3). In the US, total dry bean production and per capita availability have not changed over the last two decades (27). In some places where legumes are a central part of the diet, like Brazil, bean consumption is declining (28). Efforts to promote bean consumption in the US, such as National Bean Day on January six and National “Eat Your Beans” Day on July 3, appear to be having little impact. The sales of legumes show a seasonal pattern, with the highest expenditures during the winter. Some legume dishes, such as Hoppin' John, typically made with black-eyed pea, are served on New Year's Day, mostly in the South (29).

As a source of protein, legumes have a much lower GHG and water footprint compared with meat and dairy products and are advocated as part of sustainable diets (3). An analysis of resource requirements and environmental impact showed that production of 1 kg of protein from kidney beans required approximately 18 times less land, 10 times less water, nine times less fuel, 12 times less fertilizer, and 10 times less pesticide in comparison to producing 1 kg of protein from beef (30). Harwatt and colleagues modeled a hypothetical scenario using Life Cycle Assessment data on GHG emissions to beans and beef consumed in the US. If beans had been substituted for beef in the diet of Americans, it would have achieved ~46–74% of the GHG emissions target for 2020 and would have freed up 42% of US cropland, or nearly 700,000 km^2^ (31). In Denmark, substituting legumes for beef would reduce GHG emissions and land use by 8–12 and 5–7%, respectively (32). Adherence to plant-forward diets such as the planetary health diet recommended by the EAT-Lancet Commission (7) are considered more environmentally sustainable but can be more expensive (33). It is notable that among vegetables, beans provide the best nutritional value per penny (34).

What would be an ideal healthy dietary intake and serving size of legumes? The current US dietary guidelines recommend that those ages two and older should have a dietary pattern characterized by 0.5–3.0 cups/week of cooked beans, peas, or lentils, depending upon caloric intake (35). For example, a person with a dietary pattern of 2,000 calories/day should consume 1.5 cups/week of cooked beans, peas, or lentils, or 78 cups of cooked beans/year (34). The current per capita consumption of dry beans in the US is 3.3 kg per year (4) which is equivalent to a current per capita consumption of 15.75 cups of cooked beans/year, based upon 1.5 cups of cooked beans corresponding to 314 g of beans (36). In order to follow the US dietary guidelines, adults would need to consume roughly five times more than the current amount of 3.3 kg of dry beans to reach the recommended 16.3 kg of beans per year for an adult consuming 2,000 calories/day. A reasonable universal target for legume serving size may be 100 g of cooked pulses, equivalent to 0.5 cup (36). Given the low consumption of legumes overall, a 0.5 cup serving is a reasonable amount that is more closely aligned to the amount in a typical mixed bean dish (such as burritos, chili, etc.). It provides an average of 8 grams protein (equivalent to protein in 2 ounces of meat or poultry), 8 grams fiber (nearly 1/3 of daily recommended amount for fiber), and is a good source of potassium, magnesium, folate, calcium, iron, and complex carbohydrates (37).

It may require a great deal of behavior change to effect such a large shift in diet toward higher legume consumption following US dietary guidelines. Barriers to bean consumption, using the term “bean” in the questionnaire, include lack of knowledge about preparation or cooking of beans, concern about abdominal discomfort or flatulence, and the perception that beans are not part of a traditional diet (38). The concern about excessive flatulence from eating beans may be exaggerated. Two feeding studies of 0.5 cups/day of beans showed that only ~20% of adults reported increased flatulence after several weeks of pinto bean consumption (39). Factors that may help increase bean consumption include promotion of greater awareness of the nutritional value, taste and texture, and versatility of beans (38). The global movement, Meatless Monday, are among the public initiatives that may encourage people to try alternatives to meat such as legumes (40).

The present study has some limitations. Nielsen data are based upon retail sales only and are only indirect indicators of dietary consumption. The study did not cover purchases from the food service sector, including restaurants, or direct-to-consumer sales at farmers markets. The sales data did not cover legumes that are consumed as fresh vegetables, tofu, or mixed with other prepared foods such as stews.

In conclusion, there are differences in legume consumption and legume preferences across the US, but overall consumption of legumes remains low. A shift from animal source proteins toward a diet largely based upon plant proteins is widely advocated to optimize human health and to prevent further degradation to the planet by climate change (7). Current US dietary guidelines also recommend a higher intake of legumes. Despite the known health benefits and low cost of legumes (2, 34), increasing dietary intake of legumes remains a challenge. Further work is needed to identify barriers to increasing the consumption of legumes in the US.

## Data Availability Statement

The datasets presented in this article are not readily available because the supermarket scanning data are proprietary data obtained from Nielsen. The data are available for purchase from Nielsen in accord with their contractual agreements. NHANES data are publicly available from the National Center for Health Statistics, Centers for Disease Control and Prevention at https://wwwn.cdc.gov/nchs/nhanes. Requests to access the datasets should be directed to rdsemba@jhmi.edu.

## Author Contributions

RS, RR, VS, and MB designed the research. NR, SD, EN, and DL conducted research. VS, EN, DL, and MB provided essential materials. NR, SD, and DL analyzed data. RS, RR, VS, and DL wrote paper. RS had primary responsibility for final content. All authors have read and approved the final manuscript.

## Funding

Santa Barbara Foundation and the National Institutes of Health grant T32 HL007024.

## Author Disclaimer

Our own analyses and calculations are based in part on data reported by Nielsen through its Nielsen Service for the beans and dried beans category for the week period ending December 31, 2019, for the U.S and 31 state market and xAOC channel. Copyright © 2020, The Nielsen Company. The conclusions drawn from the Nielsen data are those of the authors and do not reflect the views of Nielsen. Nielsen is not responsible for and had no role in analyzing and preparing the results reported herein.

## Conflict of Interest

The authors declare that the research was conducted in the absence of any commercial or financial relationships that could be construed as a potential conflict of interest.

## Publisher's Note

All claims expressed in this article are solely those of the authors and do not necessarily represent those of their affiliated organizations, or those of the publisher, the editors and the reviewers. Any product that may be evaluated in this article, or claim that may be made by its manufacturer, is not guaranteed or endorsed by the publisher.
